# Synthesis, performance, and DFT insights of sustainable waste-derived S/N-doped alumina as an antiaging coating for paper sheets

**DOI:** 10.1038/s41598-026-63347-9

**Published:** 2026-07-27

**Authors:** Mona A. El-Sabour, Hebat-Allah S. Tohamy, Mohamed El-Sakhawy

**Affiliations:** Cellulose and Paper Department, National Research Centre, 33 El Bohouth Str, P.O. 12622, Dokki, Giza Egypt

**Keywords:** Alumina doped with sulfur and nitrogen, Recycling waste aluminum, Paper coating, Antiaging effectiveness, Mechanical characteristics, Flame retardancy, SEM and FTIR analysis, DFT computations, Chemistry, Engineering, Materials science

## Abstract

Using waste aluminum cans is a sustainable method created to attained alumina doped with sulfur and nitrogen as an antiaging coating for paper sheets. Acidic digestion, precipitation, calcination, and thiourea-assisted microwave doping were used to create the doped alumina, which produced nanoscale S/N-alumina particles. These particles were applied to paper sheets at different concentrations and immersion times after being mixed with a hydroxyethyl cellulose-based treatment. Mechanical testing, FTIR, SEM, flammability testing, and density functional theory calculations were used to assess the coated sheets both before and after accelerated thermal aging. Tensile strength, elongation, flame retardance, and resistance to thermal aging under accelerated conditions were all much improved by the coating, which performed best at 2% concentration and 15 min of immersion.

## Introduction

Environmental and chemical conditions have a significant impact on the long-term durability of paper, a cellulose-based substance. Over time, exposure to heat, oxygen, moisture, and acidic substances speeds up breakdown processes such acid hydrolysis and oxidation, which lessen cellulose’s degree of polymerization and encourage the creation of carbonyl and carboxyl groups. In the end, these reactions weaken the link between fibers, resulting in discoloration, embrittlement, and a loss of tensile strength^1^.

Previous studies have emphasized that thermal and oxidative aging can considerably limit the useful lifetime of paper products, making the development of protective treatments essential for cultural heritage preservation, packaging, and archival applications^2^.

To mitigate degradation, researchers have explored a variety of strengthening and protective strategies, including internal and surface sizing, polymeric coatings, and the incorporation of inorganic additives^3^. Hydrophilic polymers such as cellulose derivatives are known to enhance inter-fiber bonding by forming hydrogen-bonded networks, while fillers and metal oxides can contribute to improved durability, surface integrity, and barrier performance^4^. However, many traditional fillers either weaken the sheet at high loadings or fail to form uniform, stable interfaces with cellulose. Similarly, while several metal oxide nanoparticles have shown promise for enhancing mechanical properties, thermal resistance, and flame retardancy, their synthesis often requires expensive non-renewable precursors and energy-intensive methods that limit practical application^5^.

In recent years, the emergence of nano-alumina has attracted interest due to its high surface reactivity, thermal stability, and potential to strengthen hydrogen-bond networks within cellulose fibers^6^. These doped materials have demonstrated efficacy in increasing surface adhesion, thermal stability, and flame-retardant behavior in composite systems^7^. However, despite these benefits, little is known about their use in papermaking, especially for antiaging and fire-resistant coatings.

At the same time, there is a lot of interest in recycling and turning aluminum waste into materials with additional value due to the huge worldwide consumption of aluminum products. Recovering aluminum from used cans and turning it into valuable nanomaterials is a cost-effective and ecologically friendly way to cut waste while producing metal oxides that are suitable for industrial uses. Paper coatings that incorporate such waste-derived nanoparticles are in line with current developments in ecologically conscious, circular economy-based material creation^8^.

This study presents a novel method for creating sulfur- and nitrogen-doped alumina from used aluminum cans and using it as a protective coating for paper sheets in light of the need for efficient antiaging treatments, the shortcomings of traditional additives, and the expanding potential to upcycle aluminum waste into advanced functional materials. To encourage even dispersion and robust bonding with cellulose fibers, the doped alumina is added to a hydroxyethyl cellulose mixture. Mechanical testing is used to assess the coated papers.

This work’s main hypothesis and innovation is that sulfur and nitrogen co-doped alumina, which is made from aluminum waste using a straightforward microwave-assisted process, can create a reinforced, thermally stable, and chemically interactive coating that greatly improves the mechanical strength, resistance to thermal degradation, and flame retardancy of paper materials without sacrificing their flexibility. In order to show how the coating interacts with cellulose fibers, offering both structural reinforcement and resistance to thermal aging under accelerated conditions, this study combines waste valorization and dual doping, backed by mechanical, spectroscopic, and morphological investigations. By integrating scientific innovation with environmental effect mitigation, this scalable and relatively sustainable due to waste valorization approach provides a sustainable way to produce high-performance, long-lasting paper-based materials.

## Experiment

### Materials

The analytical-grade reagents hydroxyethyl cellulose (HEC), sodium lauryl sulfate (SLS), sodium hydroxide (NaOH), and hydrochloric acid (HCl) were purchased from Sigma-Aldrich and utilized without additional purification. Every step of the process uses distilled water.

### Paper substrate

Rakta Company (Egypt) provided the paper sheets. The sheets were composed of 60% bleached rice straw pulp, 20% bleached bagasse pulp, and 20% bleached softwood pulp. Their basis weight was 80 g/m².

### Preparation of nano-alumina from waste aluminum cans

After being properly cleaned to get rid of coatings and impurities, discarded aluminum cans were dried for 24 h at 105 °C. After being shredded into tiny pieces, the dry cans were sent to a reaction vessel.

Under a fume hood, the aluminum fragments were broken down in 5 M HCl to produce an aluminum chloride solution and hydrogen gas. After that, NaOH was gradually added while stirring constantly to neutralize the mixture. Aluminum hydroxide precipitated at pH 7–9. In order to promote the creation of nanoscale particles, a modest rate of NaOH addition was maintained.

After filtering the precipitate and repeatedly washing it with distilled water to get rid of the chloride ions, it was dried at 105 °C. To create nano-alumina, the dried aluminum hydroxide was calcined at 550 °C.

### Synthesis of sulfur- and nitrogen-doped alumina (S/N-alumina)

60 g of the produced nano-alumina, 20 g thiourea, and 40 g NaOH were combined with 200 mL of water to perform doping. After being refrigerated for the entire night, the mixture was homogenized and exposed to microwave radiation cycles in a modified domestic microwave oven operated at 100% power (750 W) for 10 min until doping was finished. Number of cycles involves 10 on/off cycles of 60 s (1 min of irradiation followed by a 30-second cooling window) at approximately 100 °C.

S/N-doped alumina was the end product.

### Preparation of the coating suspension

HEC (3% w/v) was thoroughly dispersed by vigorously swirling it in distilled water. SLS (15% of the mass of alumina) and S/N-alumina were added to the polymer solution.

To create a stable, uniform suspension, the mixture was vigorously mixed and then sonicated for 30 min. S/N-alumina was effectively dispersed throughout the coating formulation thanks to the combined stirring and sonication.

### Coating procedure

Paper sheets were heated to 90 °C for six hours prior to coating in order to eliminate any remaining moisture and activate the fiber surface. For chosen immersion periods ranging from 0 to 20 min, paper samples were submerged in the coating suspension at concentrations of 0–2% (w/v). S/N-alumina diffused into the fiber network and settled on the paper’s surface during immersion. The sheets were pressed between absorbent papers to remove the surplus solution after immersion. In order to facilitate water evaporation and the development of an inorganic-polymeric coating structure, samples were subsequently dried at 40 °C to constant weight. Before testing, all samples were conditioned for a full day at 23 °C and 50% relative humidity.

### Weight% gain (WPG)

WPG was calculated based on the oven-dried weights of the uncoated (Wx) and coated (Wy) papers:

1 $$\:\mathrm{W}\mathrm{P}\mathrm{G}\:\mathrm{\%}=\frac{\mathrm{W}\mathrm{y}-\mathrm{W}\mathrm{x}}{\mathrm{W}\mathrm{x}\:}\times\:100$$

### Accelerated aging procedure

Accelerated aging was performed by placing samples in an oven at 140 °C for 2 h. Mechanical, flammability, and structural analyses were conducted before and after aging to evaluate the antiaging performance of the coatings.

### Mechanical testing

Tensile strength and elongation were measured according to TAPPI T494-06 using a Universal Testing Machine (Model 4201). Young’s modulus was calculated from the tensile data. Samples were cut and tested in accordance with standard requirements. All mechanical measurements were performed in triplicate (*n* = 3), and the reported values represent the mean ± standard deviation. Statistical variability was calculated to assess the reproducibility and reliability of the experimental results.

### Flammability testing

Flame retardance was evaluated using a CSI oxygen index meter (Atlas) following ASTM D2863. Samples (100 × 52 × 0.3 mm) were tested to determine the limiting oxygen index (LOI), described as the lowest concentration of oxygen needed to maintain combustion.

### Spectroscopic XRD and microscopic characterization

#### FTIR analysis

Using KBr pellets and a Mattson 5000 spectrometer, FTIR spectra were captured.

The average hydrogen bond strength was estimated from the ratio of O–H and C–H absorption bands via Eq. (2).

2 $$\:\mathrm{M}\mathrm{H}\mathrm{B}\mathrm{S}=\frac{{A}_{OH}}{{A}_{CH}}$$

where the FTIR absorbance of the OH and CH peaks is denoted by AOH and ACH, respectively.

#### X-ray diffraction (XRD) analysis

The crystalline structure and phase composition of the synthesized undoped alumina (AL) and S/N co-doped alumina (SN) nanoparticles were characterized by X-ray diffraction (XRD) using a Rigaku diffractometer equipped with a D/teX Ultra 250 detector and Cu Kα radiation (λ = 1.5406 Å), operated at an accelerating voltage of 40 kV and a current of 30 mA. Diffraction patterns were recorded over a 2θ scan range of 5° to 80° using a θ/2θ scan axis in one-dimensional scan mode, with a step width of 0.03° and a scan speed of 30°/min. Prior to analysis, the samples were finely ground and uniformly spread on the sample holder to obtain a smooth surface. The recorded diffraction profiles were analyzed for phase identification by comparison with standard reference cards (ICDD database), and crystallite sizes were estimated from the full width at half maximum (FWHM) of the diffraction peaks using the Scherrer equation: D = Kλ/β cosθ, where D is the mean crystallite size, K is the shape factor (0.9), λ is the X-ray wavelength, β is the FWHM in radians, and θ is the Bragg diffraction angle.

#### SEM imaging

A Quanta/250-FEG scanning electron microscope running at 30 kV was used to analyze surface morphology.

### DFT calculations

Gaussian 09 W was used to calculate density functional theory (DFT) using the B3LYP functional and the 6-31G(d) basis set. To interpret interactions between the coating and cellulose fibers, geometry optimization and electronic structure parameters (performed using the Berny algorithm) were assessed, including total energy (ET), the energy of the highest occupied MO E_HOMO_, the energy of the lowest unoccupied MO E_LUMO_, the energy gap (Eg), the dipole moment (µ), the absolute hardness (η), the absolute softness (σ), and the chemical softness (S) (Eqs. 3–6)^9^.

3 $$\:{E}_{gap}=({E}_{LUMO}-{E}_{HOMO})$$

4 $$\:{\upeta\:}=\frac{({E}_{LUMO}-\:{E}_{HOMO})\:\:}{2}\:$$

5 $$\:{\upsigma\:}=\frac{1\:\:}{{\upeta\:}}$$

6 $$\:\mathrm{S}=\frac{1\:\:}{2{\upeta\:}}$$

## Results and discussions

### FTIR spectroscopy of S/N-alumina

**Fig. 1:**
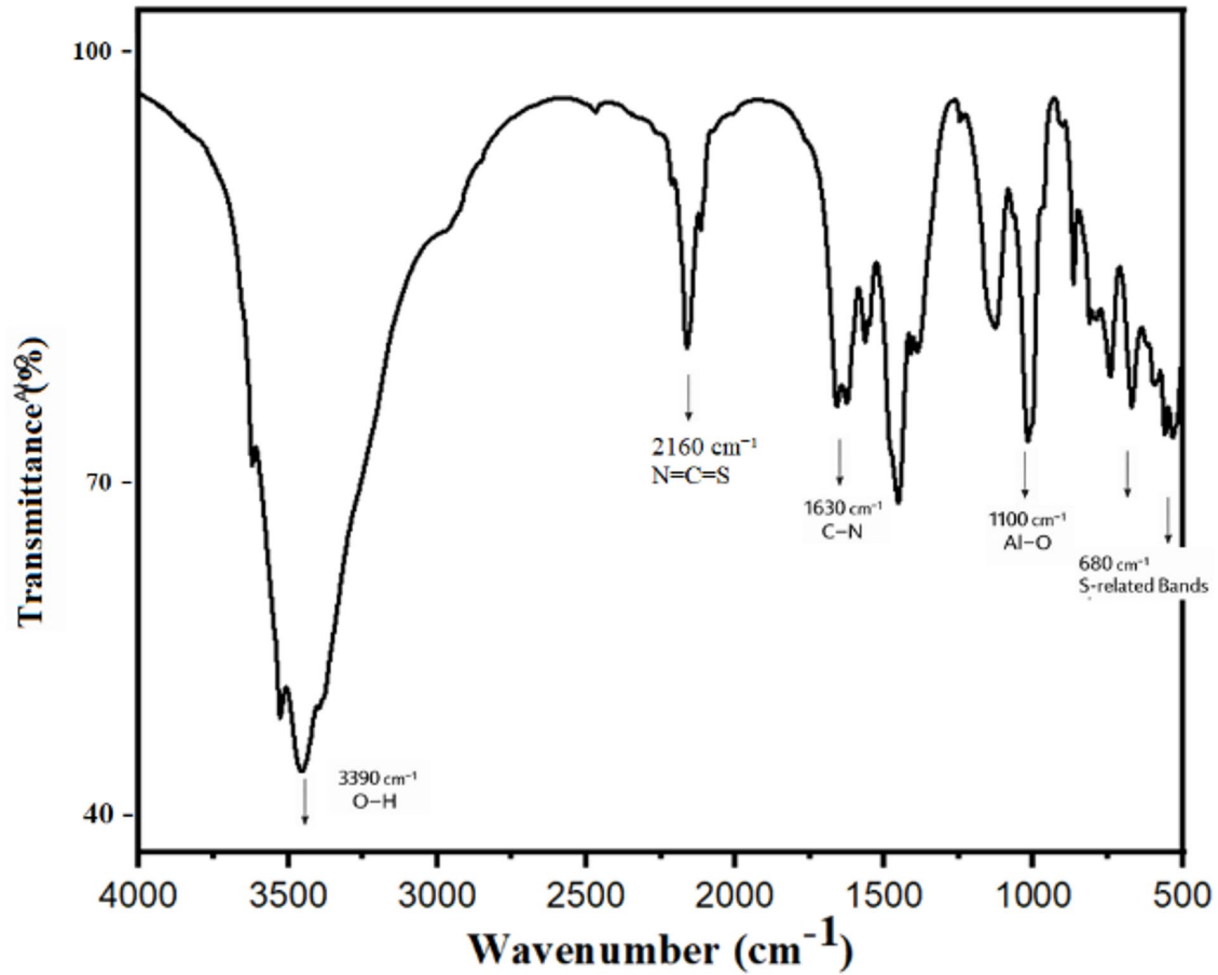
FTIR of S/N-alumina.

The FTIR spectrum of the S/N-doped alumina, Fig. 1, exhibits several characteristic vibrational features that collectively confirm both the successful formation of alumina and the incorporation of sulfur and nitrogen heteroatoms into the structure. The broad absorption band in the range of 3200–3600 cm⁻¹, typically associated with O–H stretching, indicates the presence of surface hydroxyl groups^10^. These –OH groups are known to contribute to high surface reactivity and play a critical role in hydrogen-bonding interactions with cellulose fibers once applied as a coating. Their presence is also indicative of partial surface hydration, which is common for nanoscale alumina materials.

The sharp, distinct absorption band appearing at approximately 2160 cm⁻¹ is attributed to the asymmetric stretching vibration of isothiocyanate or thiocyanate groups. Additional bands at approximately 1250–1400 cm⁻¹ and 1500–1650 cm⁻¹ can be attributed to C–N stretching and N–H bending modes originating from thiourea decomposition intermediates^11^. These signatures serve as direct evidence of nitrogen incorporation. Similarly, modest absorptions in the 1040–1150 cm⁻¹ range are associated with C–S vibrations or S = O stretching, indicating sulfur-related species attached to the alumina surface^12^.

In the 500–900 cm⁻¹ region, strong Al–O and Al–O–Al lattice vibrations characteristic of γ-alumina is clearly present^13^. The retention of these peaks confirms that thiourea-assisted doping did not disrupt the fundamental crystalline oxide framework. Instead, doping is likely confined to surface modifications or substitutional positioning within defect sites.

A chemically enriched surface that can encourage multipoint contacts, hydrogen bonding, electrostatic attraction, and Lewis acid–base interactions with cellulose is suggested by the coexistence of O–H, Al–O, C–N, and S-related functions. Strong adherence, consistent deposition, and fiber consolidation seen in the coated paper samples are explained by this increased chemical activity^14^.

### XRD analysis

**Fig. 2:**
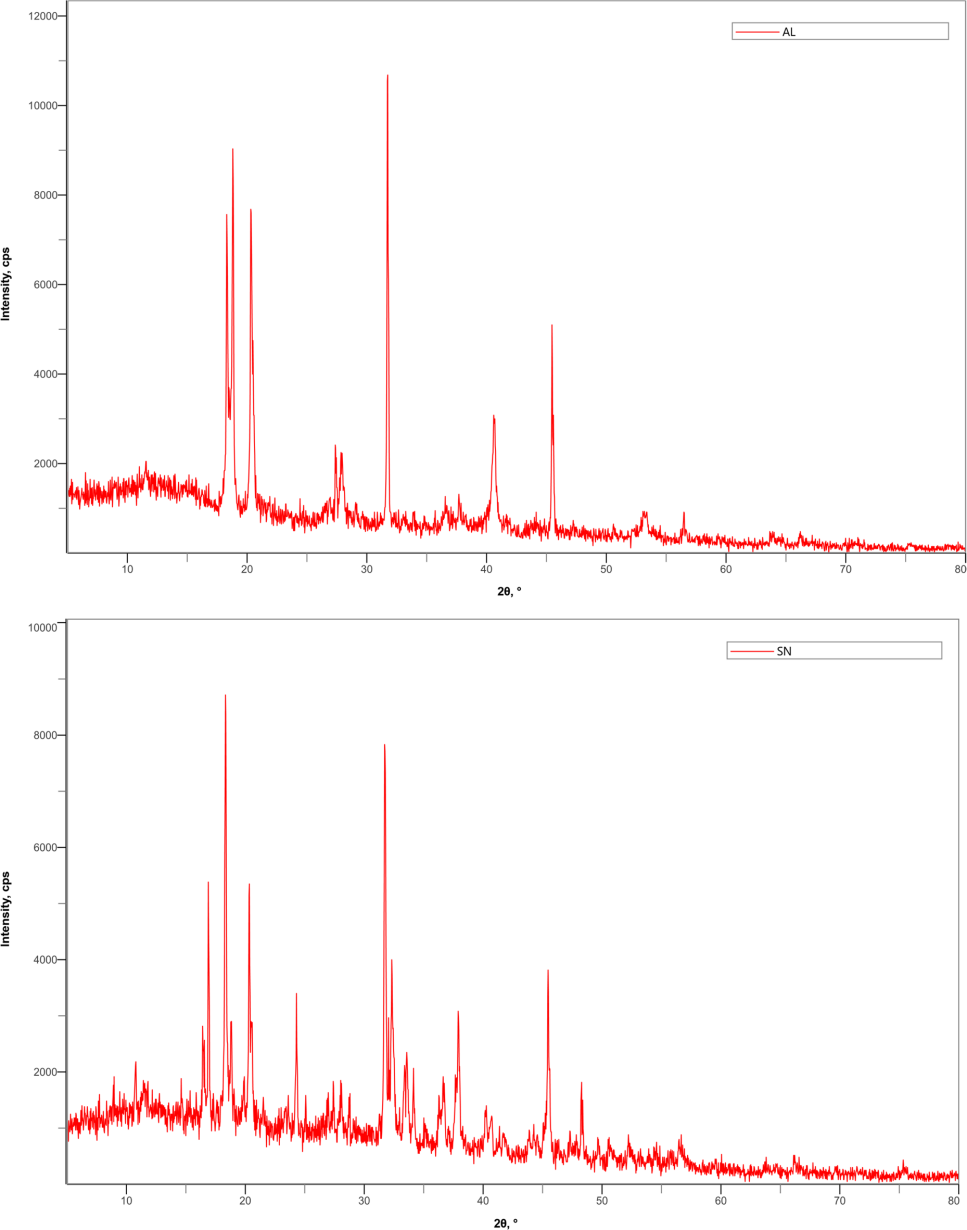
XRD patterns of the undoped alumina (AL) and S/N co-doped alumina.

The XRD patterns of the undoped alumina (AL) and S/N co-doped alumina (SN) nanoparticles are presented in Fig. 2. The diffraction pattern of the synthesized alumina nanoparticles exhibited characteristic reflections at 18.32°, 31.73°, 37.73°, and 45.44°, corresponding to the (111), (220), (311), and (400) planes of γ-Al₂O₃ (JCPDS 10–0425), confirming successful formation of the γ-alumina phase. The broad diffraction peaks, evidenced by large full width at half maximum (FWHM) values exceeding 0.5°, indicate nanocrystalline nature and moderate crystallinity. The average crystallite size calculated by the Scherrer equation was approximately 96 nm. Upon S/N doping, slight shifts of the 31.7° peak and broadening of the FWHM suggesting successful dopant incorporation into the alumina lattice and increased structural defects. Additional minor peaks in the doped sample were attributed to secondary S- and N-containing phases co-formed during synthesis.

### DFT calculations

DFT investigations provide important theoretical insights into the electronic structure and reactivity of the S/N-doped alumina when compared to untreated cellulose surfaces.

**Fig. 3:**
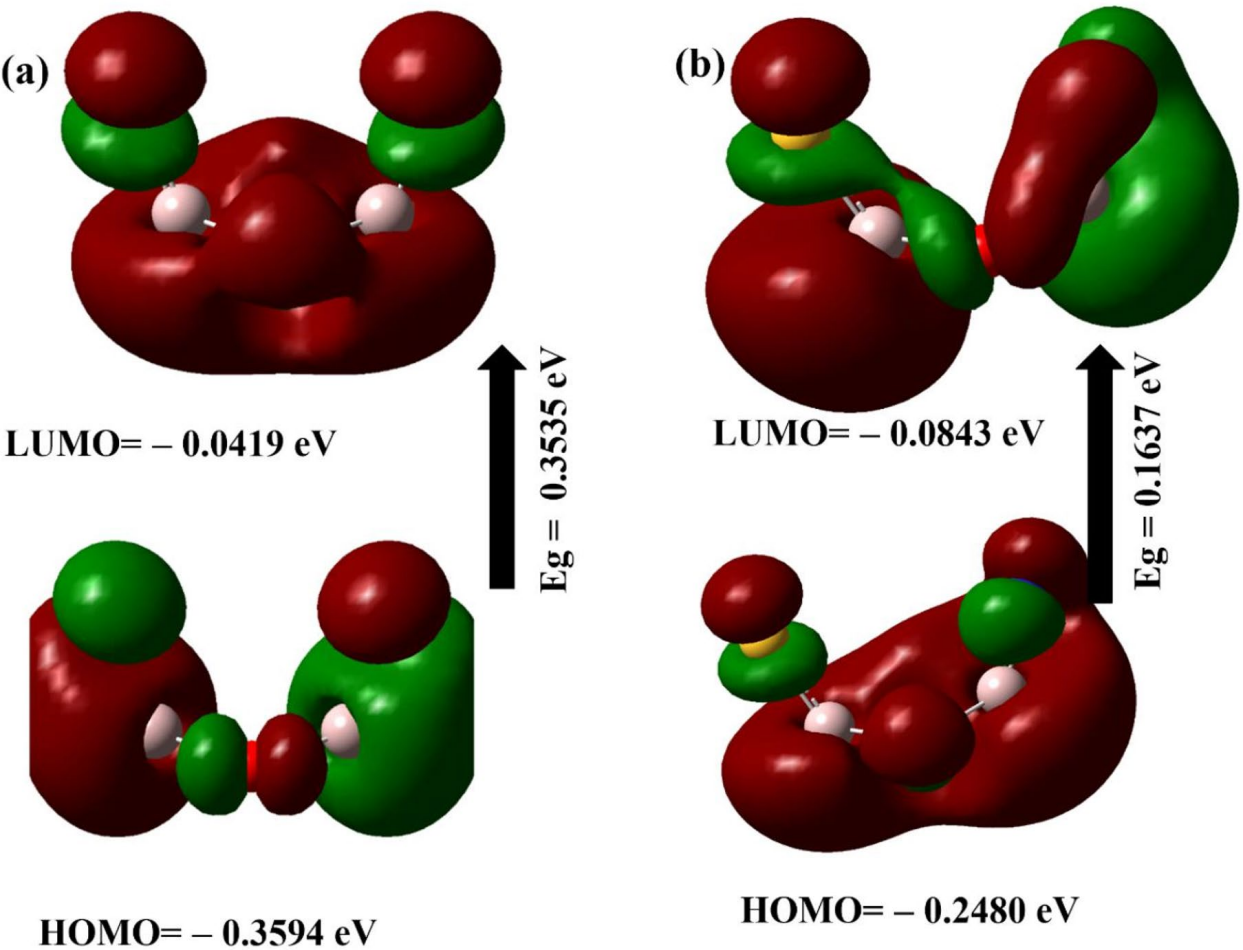
DFT calculations for (**a**) blank paper and (**b**) S/N-alumina coated paper.

According to Fig. 3; Table 1, the calculated HOMO–LUMO gap (Eg) for the doped material decreases in comparison to pristine cellulose, indicating evident improved charge-transfer capability and increased electronic softness^15^. In line with the FTIR results indicating more functional groups, this decrease in Eg indicates that the modified alumina has a more chemically sensitive surface.

**Table 1:** The quantum chemical parameters.

DFT	Blank paper	Nitrogen/sulfur aluminate
E_LUMO_ (eV)	–0.0419	–0.0843
E_HOMO_ (eV)	–0.3594	–0.2480
E_g_ (eV)	0.3535	0.1637
E_T_ (au)	–1208.88	–1005.15
µ (Debye)	12.63	4.83
ɳ (eV)	0.1768	0.8185
σ (eV)	5.6561	6.0187
Pi (eV)	4.08	4.52
S (eV)	2.8281	3.0544

The development of highly polar interactions at the interface is indicated by an increase in the coated system’s dipole moment (µ). In addition to potential Lewis acid–base interactions mediated by doped nitrogen and sulfur atoms, these interactions most likely entail hydrogen bonding between surface O–H groups on alumina and hydroxyl groups in cellulose.

The electronic softness (σ) and hardness (η) values further clarify this relationship. The higher hardness of the S/N-doped alumina (η = 2.22 eV) confirms a rigid, stable crystalline environment, while the cellulose’s lower hardness indicates greater polarizability. Essentially, the S/N-doped alumina acts as a stable, hard anchor, while the cellulose behaves as a more flexible partner. This electronic complementarity is ideal for forming a robust, reinforced interface. Furthermore, we examined the µ, which quantifies the polarity of each structure. The high dipole moment of the cellulose (12.63 Debye) suggests it is a highly polar, hydrophilic substrate, ready to engage in hydrogen bonding. Our S/N-doped alumina, with its specific dopant distribution, is chemically tuned to match these polar sites. The shift in chemical potential (Pi) and global softness (S) corroborates that the S/N-doped alumina is not merely an inert filler; it is an electronically active species^16^.

The consistent improvements in mechanical properties seen in experiments are in close agreement with these theoretical expectations. They corroborate the finding that S/N-alumina creates electronic compatibility with cellulose, resulting in improved thermal stability, decreased oxidative vulnerability, and improved fiber cohesiveness. The DFT results provide qualitative support for the observed interfacial interactions, but do not directly establish a quantitative relationship with macroscopic mechanical performance.

### Morphological observations (SEM)

**Fig. 4:**
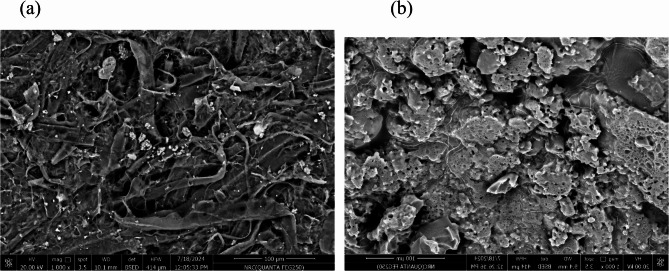
SEM analysis of (**a**) blank paper and (**b**) paper coated with S/N-alumina.

The SEM micrographs (Fig. 4) reveal significant morphological transformation upon application of the S/N-doped alumina coating. The uncoated paper shows loosely packed, highly porous fiber structures with visible gaps and irregular voids, features typical of unfilled lignocellulosic networks. These voids make the paper susceptible to moisture penetration, mechanical deformation, and accelerated aging. After coating, SEM images show that S/N-alumina particles are uniformly distributed across fiber surfaces, forming a continuous inorganic–polymeric layer. The nanoparticles appear to embed themselves within surface irregularities, effectively “bridging” adjacent fibers. This inter-fiber bridging reduces porosity, enhances sheet densification, and limits pathways for oxygen and heat transfer.

Furthermore, the coating creates smoother, more cohesive surfaces by securely binding fibrillar structures and filling tiny gaps. The observed gains in elongation and tensile strength, as well as the decreased deterioration following thermal age, are explained by this morphological reinforcement.

Flame retardancy is another benefit of the enhanced microstructure. While doped materials encourage the production of char under heat, the coating prevents direct contact between cellulose and oxygen by creating an inorganic barrier layer^17^. Thus, the SEM d S/N-alumina coating’s strong adherence and efficient layer formation.

### Weight% Gain (WPG)

**Fig. 5:**
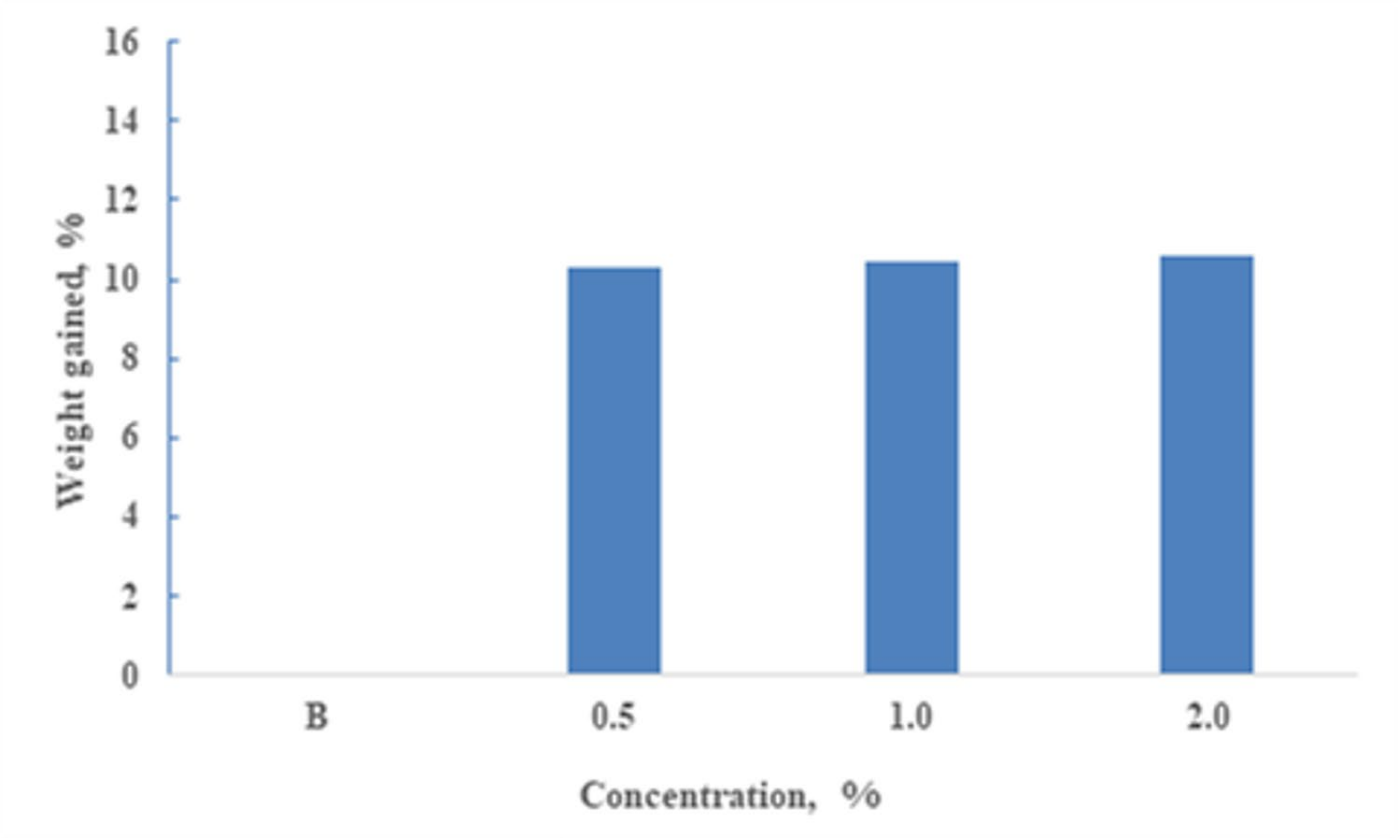
The weight gained percent of coated paper with S/N-alumina vs. concentration at 2 min. immersing time.

**Fig. 6:**
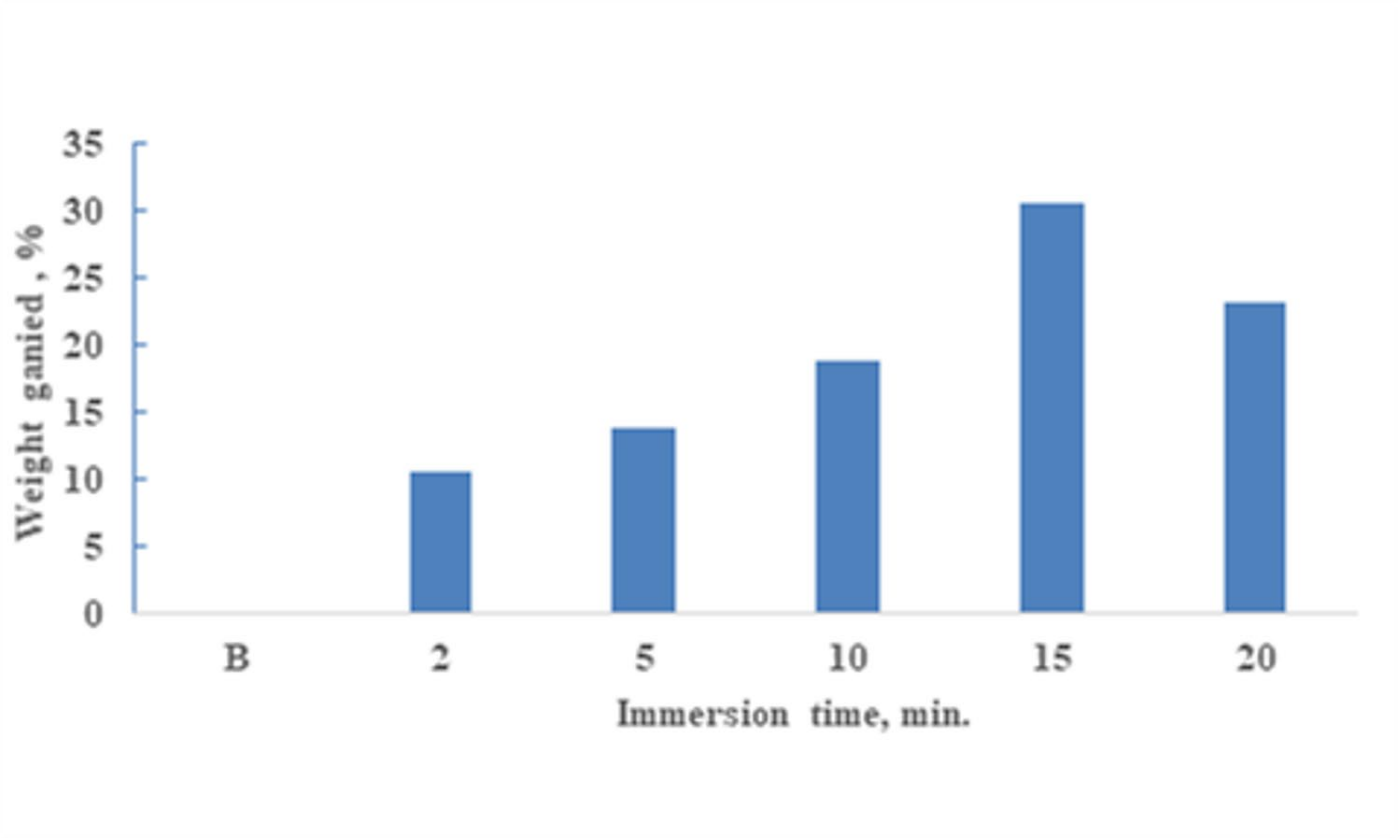
The weight gained percent of coated paper with S/N-alumina vs. immersion time at 2% concentration.

Material uptake during coating was measured using WPG. Effective deposition at higher concentrations is confirmed by Figs. 5 and 6, which demonstrate that raising the concentration of S/N-doped alumina from 0 to 2% produced a WPG of 10.61%.

Likewise, increasing immersion time from 0 to 15 min increased WPG to 30.52%. Beyond 15 min, the WPG decreased, which can be attributed to the excessive swelling of the cellulosic fibers in the aqueous suspension and oversaturation effect where previously deposited material partially redissolves, detaches, or becomes displaced due to prolonged exposure. Moreover, prolonged immersing may cause partial disintegration or weakening of the fiber structure, which decreases the paper’s ability to retain the coating material effectively. This behavior reflects a saturation–desorption equilibrium typical in porous fibrous substrates^18^.

### Mechanical properties

Mechanical properties of paper describe how the sheet behaves when forces are applied to it. These properties are essential for evaluating paper performance during end-use applications. Key mechanical properties include tensile strength which is defined as the maximum stress that a sheet of paper can withstand while being stretched before it breaks. Tensile strength reflects the strength of the fiber network and depends on fiber type and bonding between fibers, refining degree, paper density, and surface treatment, while high moisture or excess fillers reduce it. High tensile strength means the paper is durable, well bonded, and suitable for applications requiring mechanical stability.

#### Effect of concentration

**Fig. 7:**
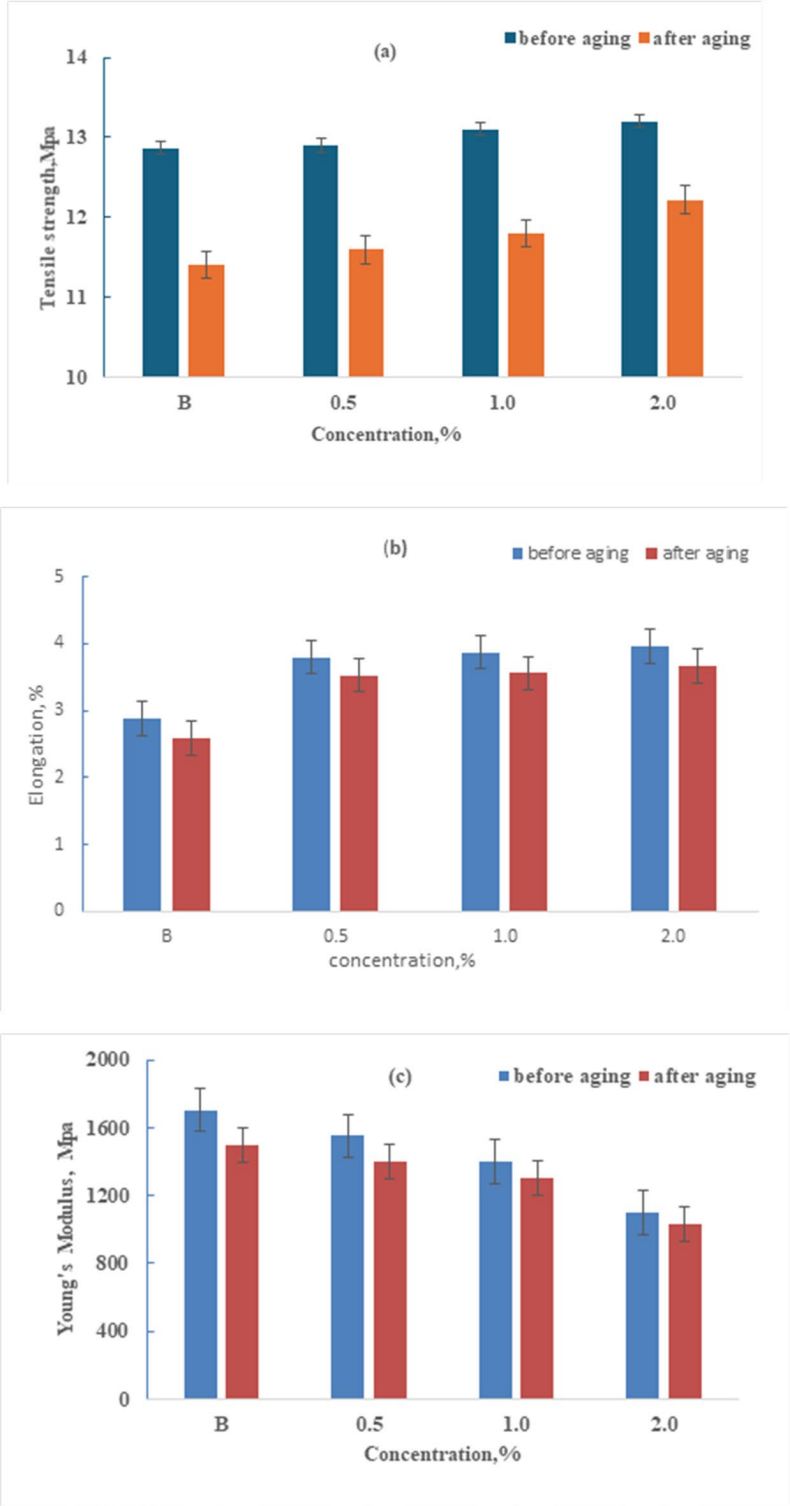
The mechanical properties of coated paper with S/N-alumina at different concentrations before and after aging (**a**) tensile strength, (**b**) elongation, (**c**) young’s modulus. (Error bars represent standard deviation (*n* = 3), indicating the reproducibility of the measurements.).

Mechanical testing demonstrated an evident improvement in mechanical properties of the coated paper before aging as the S/N-alumina concentration increased, as shown in Fig. 7. At 2% concentration, tensile strength increased to 13.2 MPa and elongation to 4.42%, whereas Young’s modulus showed a gradual decline, reflecting increased flexibility. These enhancements derive from the formation of a uniform inorganic–polymeric network that strengthens fiber–fiber bonding and reduces inter-fiber slippage^19^.

#### Effect of immersion time

**Fig. 8:**
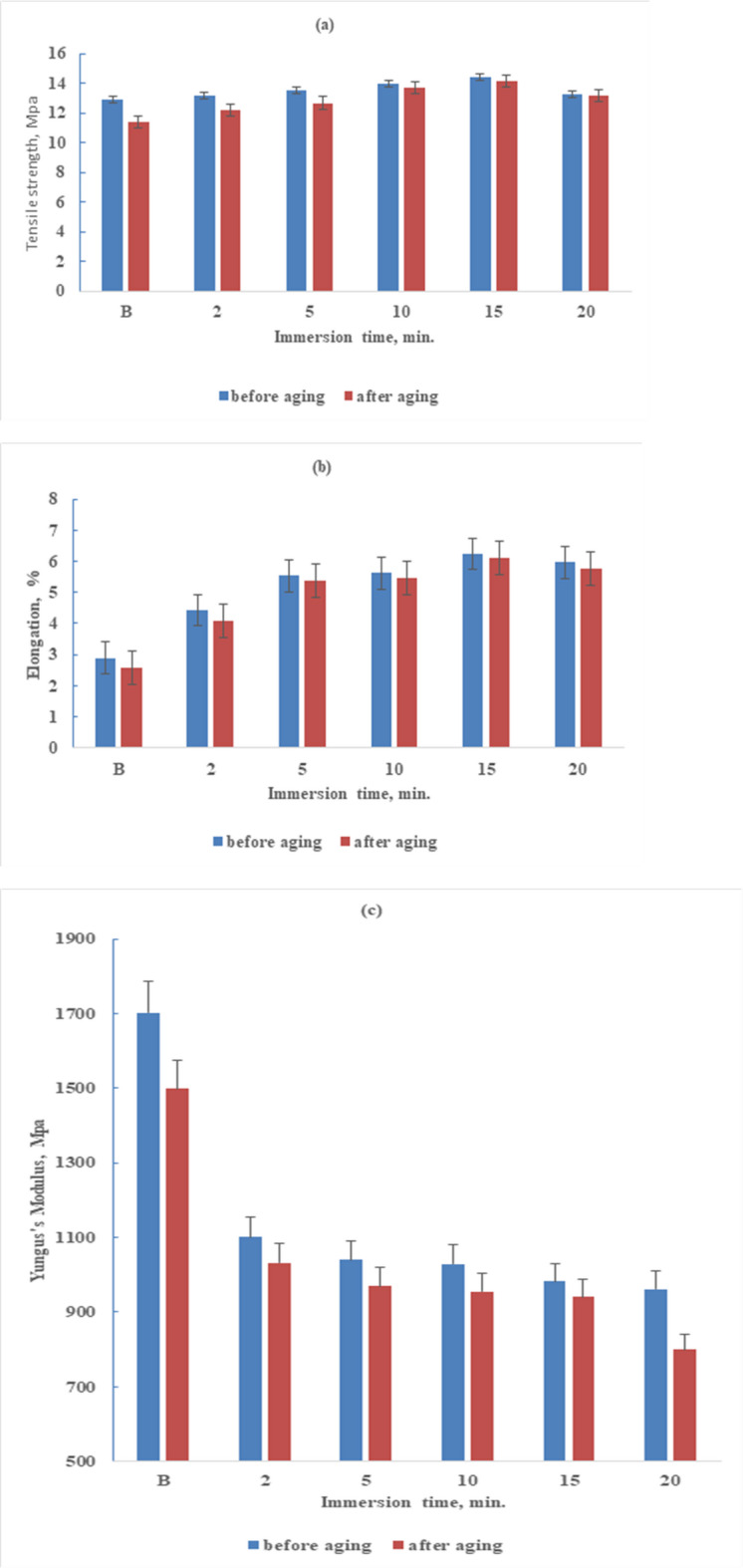
The mechanical properties of coated paper with S/N-alumina before and after aging at different immersion times (**a**) tensile strength, (**b**) elongation, (**c**) young’s modulus. (Error bars represent standard deviation (*n* = 3), indicating the reproducibility of the measurements.)

Figure 8 shows that, as the immersion time increased, mechanical properties improved up to 15 min, reaching tensile strength of 14.38 MPa and elongation of 6.23%. However, the young’s modules exhibit a gradual decline. This enhancement is mainly attributed to increase in the quantity of coated materials (weight gain), deposited on the paper surface as a result of the prolong immersing time, which produced a thicker and more uniform layer that contributed to the improvement of mechanical properties^20^.

The mechanical properties reached a plateau beyond 15 min, with only minor variations within experimental deviation, indicating a saturation behavior in coating effectiveness likely results from excessive deposition that disrupts fiber bonding or creates brittle agglomerates^21^. The optimal immersion time thus balances coating thickness with the preservation of fiber flexibility. Hydroxyethyl cellulose, included in the coating formulation, contributed additional binding through film formation around the fibers. Its structural similarity to cellulose supports hydrogen bonding, complementing the reinforcing effect of the S/N-doped alumina^22^. The relatively small standard deviations observed across all measurements (*p* < 0.05) confirm the good reproducibility of the coating process and the reliability of the mechanical performance trends.

### Aging behavior of coated paper

Paper aging is a progressive deterioration process mainly caused by acid hydrolysis, oxidation of cellulose and in some cases photochemical and thermal degradation. The aging of paper refers to the gradual chemical and physical deterioration of cellulosic fibers, which results in yellowing and loss of mechanical strength.

Coating plays a protective role by acting as a barrier against environmental impact, reducing the influence of moisture, light, and chemical pollutants. Thus, surface treatments and coatings can significantly slow degradation and improve the long-term permanency of paper materials^23^.

Accelerated aging at 140 °C for 2 h caused mechanical deterioration in all samples, but the coated papers with S/N-alumina retained significantly more strength than the untreated controls, as shown in Figs. 7 and 8. The untreated paper (B) exhibited a decrease of 11.34%, 10.42%, and 11.97% in tensile strength, elongation, and Young’s modulus, respectively.

In contrast, the coated papers showed markedly smaller declines. At 2 min immersion time and 2% concentrations, the percent decrease in mechanical properties for aged, coated paper is 7.42, 7.24, 6.22% for tensile strength, elongation and young’s modules respectively. At 15-minute immersion, decreases were only 1.53% in tensile strength, 2.08% in elongation, and 4.17% in Young’s modulus. This demonstrates that the coating effectively mitigated thermal degradation.

The clear improved resistance to aging is attributed to the protective barrier formed by the S/N-doped alumina. This barrier reduces oxidative and hydrolytic reactions by limiting oxygen access and stabilizing the cellulose structure^22,24^. Additionally, doping elements likely enhance thermal resistance and modify the degradation pathway, resulting in less severe structural damage, which indicates that the treatment of paper with S/N-alumina could improve the thermal aging resistance under accelerated conditions.

### Flammability performance (LOI)

Limiting Oxygen Index (LOI) is defined as the minimum concentration of oxygen, expressed as a percentage, in a mixture of oxygen and nitrogen that is required to just support the combustion of a material under specified test conditions. The material with higher LOI values is less flammable, while those with lower LOI values burn more easily. It is widely used in evaluating the fire resistance of polymers, coatings, and composite materials.

**Table 2:** S/N-alumina-coated paper sheets’ flame retardance at varying concentrations.

Immersing Time, min	conc., %	LOI, vol%
0	0	18.0
2	0.5	18.5
2	1.0	18.7
2	2.0	19.0

**Table 3:** S/N-alumina-coated paper sheets’ flame retardance at different immersion times and concentrations.

Immersing Time, min	conc., %	LOI, vol%
0	0	18.0
2	2	19.0
5	2	19.1
10	2	20.5
15	2	22.5
20	2	21.5

The limiting oxygen index (LOI) of the untreated paper was 18.0 vol%, indicating high flammability. Coated samples displayed increasing LOI values with both higher concentration and longer immersion times, Tables 2 and 3, reaching 19.0 vol% at 2% concentration and 22.5 vol% at 15 min. These results indicate improved flame retardancy based on limiting oxygen index (LOI) measurements, which results from the development of an inorganic barrier that limits oxygen transport and heat transmission^23,25^; however, the values remain below the threshold typically associated with self-extinguishing materials. Uniform dispersion of the oxide was promoted by the polymeric component (HEC), allowing for synergistic improved flame retardancy without sacrificing mechanical qualities^26,27^.

Because a protective barrier that restricts heat transfer and oxygen diffusion to the paper surface is formed, paper coated with S/N-alumina shows improved flame retardancy based on LOI. When exposed to elevated temperatures, these coatings may experience thermal degradation, produce non-flammable gases or form a stable char layer that insulates the underlying cellulose fibers and stops additional combustion. This protective mechanism increases the paper’s heat stability and decreases its flammability, suggesting that the applied coating successfully increases the flame retardancy^28^.

### Integrated interpretation

The combination of structural, morphological, mechanical, theoretical, and flammability findings confirms that sulfur- and nitrogen-doped alumina significantly enhances paper durability. The modified alumina interacts strongly with cellulose, reinforcing the fiber network while improving thermal and flame retardancy. The waste-derived origin of the alumina also underscores the environmental and economic value of the approach. Optimal performance was achieved with 2% concentration and a 15-minute immersion time, where mechanical properties, thermal stability, and flame retardancy were maximized.

### Comparison with previous studies

As shown in Table 4, the present study demonstrates a competitive improvement in mechanical properties compared to previously reported coating systems, while uniquely utilizing waste-derived alumina as a functional additive. Although the achieved LOI values remain below the threshold for full flame retardancy, the combined enhancement in tensile strength, elongation, and thermal aging resistance highlights the effectiveness of the S/N-doped alumina coating. Importantly, this work distinguishes itself through its integration of waste valorization and dual heteroatom doping within a cellulose-compatible polymer matrix.

**Table 4:** Comparison of the present study with previously reported paper coating systems.

Study	Coating Material	Source of Material	Key Additives / Modification	Tensile Strength Improvement	Elongation Improvement	LOI (vol%)	Key Functional Outcomes	Remarks
^19^	Nanofiber-based coatings	Synthetic nanomaterials	Post-treatment strategies	Significant increase (~ 20–30%)	Moderate increase	Not reported	Improved mechanical durability	Complex processing
^20^	CaCO₃ + fatty acid	Mineral filler	Hydrophobic modification	Moderate increase	Improved flexibility	Not reported	Enhanced water resistance	No fire-retardant functionality
^23^	Gelatin coating	Natural biopolymer	Protein-based treatment	Moderate increase (~ 10–15%)	Slight improvement	Not reported	Improved thermal aging resistance	Limited flame retardancy
^25^	Polymer flame-retardant composites	Synthetic polymers	Advanced flame retardants	Variable	Variable	> 25	High fire resistance	Not paper-specific
^26^	Flame-retardant polymer coating	Synthetic chemicals	Phosphorus-containing additives	Moderate increase	Slight decrease	~ 24–28	Strong flame retardancy	Uses non-renewable chemicals
This Study	S/N-doped nano-alumina + HEC	Waste aluminum cans	Dual doping (S, N) + biopolymer matrix	Significant increase (~ 12–18%)	Significant increase (~ 30–100%)	Up to 22.5	Improved mechanical strength, thermal aging resistance, and LOI	Sustainable feedstock; moderate flame retardancy

## Conclusions

Sulfur- and nitrogen-doped alumina synthesized from waste aluminum cans proved to be an effective coating material for enhancing the performance and long-term stability of paper sheets. The coating increased tensile strength and elongation, with the highest improvements observed at 2% concentration and a 15-minute immersion time. FTIR and SEM analyses confirmed strong interactions between the coating and cellulose fibers, while the weight-gain measurements indicated efficient deposition of the nano-oxide layer. After accelerated thermal aging, the coated papers exhibited markedly lower reductions in mechanical properties compared with uncoated samples, demonstrating the coating’s capacity to mitigate cellulose degradation. The flame-retardant performance also improved, with limiting oxygen index values rising significantly for treated papers.

These results highlight the potential of S/N-alumina as a relatively sustainable, waste-derived material that enhances both the mechanical integrity and resistance to thermal aging under the tested conditions of paper. Limitations of the current study include the use of a single aging condition and the absence of long-term natural aging evaluations. More definitive structural and compositional techniques such as XRD and XPS or high-resolution elemental mapping are required to confirm γ-alumina phase purity and distinguish between lattice doping and surface functionalization. In addition, TGA was not conducted, limiting detailed interpretation of thermal degradation pathways and char formation mechanisms. Finally, the density functional theory (DFT) calculations were performed using a simplified model system that does not explicitly account for the HEC binder or surfactant environment, and therefore provide qualitative rather than fully representative insight into interfacial interactions.

Future work should investigate the coating’s performance under varying environmental stresses, explore scaling the process for industrial application, and examine interactions with different paper types and coating formulations.

## Data Availability

All data generated or analysed during this study are included in this published article.
